# Evaluating qigong as integrative support for COVID-19 and Long-COVID-19 rehabilitation: a systematic review

**DOI:** 10.3389/fpsyg.2024.1403130

**Published:** 2024-05-14

**Authors:** Michele Antonelli, Davide Donelli

**Affiliations:** ^1^Department of Public Health, Azienda Unitá Sanitaria Locale – Istituto di Ricovero e Cura a Carattere Scientifico of Reggio Emilia, Reggio Emilia, Italy; ^2^Cardiology Unit, University Hospital of Parma, Parma, Italy

**Keywords:** qigong, mind-body therapy, COVID-19, coronavirus, health

## Abstract

**Introduction:**

Amidst the ongoing global impact of COVID-19 on public health, there is an increasing focus on holistic strategies encompassing integrative therapies and rehabilitation techniques, particularly in addressing the challenges posed by Long-COVID-19. This review investigates the potential of Qigong, an ancient Chinese practice characterized by gentle movements, controlled breathing, and meditative elements, within the context of COVID-19.

**Methods:**

A systematic search of PubMed, EMBASE, Web of Science, Scopus, and Google Scholar was conducted to identify pertinent clinical studies.

**Results:**

Following thorough database scrutiny, nine studies were identified as meeting the eligibility criteria. Across the spectrum of COVID-19 severity, individuals engaging in qigong practice exhibited notable enhancements in both physical and psychological wellbeing, evidenced by ameliorated respiratory symptoms, reduced anxiety levels, enhanced sleep quality, bolstered mental wellbeing, and augmented health-related quality of life. Moreover, qigong training, whether employed independently or in conjunction with other therapies, demonstrated beneficial effects on Long-COVID-19 symptoms, encompassing persistent respiratory issues, dizziness, sleep disturbances, and compromised health-related quality of life.

**Discussion:**

This review underscores the necessity for further investigation to quantify and standardize the contribution of Qigong to COVID-19 recovery and rehabilitation. Such endeavors aim to integrate this accessible and low-impact practice into public health strategies and comprehensive treatment regimens.

**Systematic review registration:**

The review protocol was registered in the Open Science Framework under the following doi: 10.17605/OSF.IO/7K5X6 (URL: https://osf.io/7k5x6).

## 1 Introduction

### 1.1 Long-COVID-19

COVID-19, caused by the SARS-CoV-2 virus, emerged as a pandemic from early 2020, with significant global consequences not only on health but also on socio-economic aspects (Ciotti et al., 2020; Mishra et al., 2020). While the disease typically manifests with fever, cough, and predominantly respiratory symptoms, it can result in severe interstitial pneumonia characterized by an abnormal systemic inflammatory response, sometimes leading to fatal outcomes despite hospital care, especially in individuals with pre-existing comorbidities (Çalica Utku et al., 2020). Research conducted on recovered patients has revealed that COVID-19, even in its non-severe manifestations, can result in persistent symptoms that endure or emerge over a span of 3 months following the acute phase of the disease, aligning with the current definition of Long-COVID-19 (Crook et al., 2021). These symptoms encompass a spectrum of manifestations, including chronic fatigue, headache, cardiac or respiratory alterations, arthralgia, muscle pain, taste or olfactory dysfunctions, sleep disorders, cognitive impairment, post-traumatic stress disorder, and mood disturbances, which may persist for weeks or even months following the initial illness episode (Sykes et al., 2021; Yong, 2021). In particular, Long-COVID-19 is defined by the WHO-led Delphi study as symptoms persisting for at least 3 months from the onset of the acute disease, with manifestations lasting at least 2 months, often presenting in an episodic nature, while clinicians lack standardized guidelines for diagnosis, relying on symptom evaluation and exclusion of other health conditions (Srikanth et al., 2023). Elderly age, female gender, pre-existing comorbidities, severe COVID-19 requiring hospitalization, and supplemental oxygen are all risk factors associated with the development of Long-COVID-19 (Srikanth et al., 2023). The distinction between the acute phase and Long-COVID-19 highlights the evolving trajectory of the disease's effects on individuals, emphasizing the need for tailored therapeutic strategies. In the results section of this review, studies investigating the effects of qigong specifically for COVID-19 and those focusing on Long-COVID-19 will be differentiated, reflecting the specific considerations required for managing the diverse manifestations of the disease over time.

Despite the fact that vaccines have proven effective in decreasing the occurrence of COVID-19 and mitigating its prolonged symptoms (Català et al., 2024), evidence-based rehabilitation strategies designed to expedite recovery from the disease are still useful, along with proper treatment management, addressing comorbidities, and compensating for socio-economic disparities in access to healthcare. Additionally, proper treatment management and efforts to bridge socio-economic gaps in healthcare access are essential components in this endeavor (Hossain et al., 2023). Among various approaches, mind-body techniques stand out for their dual benefit, as they simultaneously alleviate physical and mental symptoms by engaging both aspects of the human experience (Astin et al., 2003). In fact, the potential utility of mind-body interventions in post-COVID recovery is underscored by their holistic approach to health, which does not isolate physical symptoms from mental wellbeing (Maric et al., 2021; Alschuler et al., 2022; Brough et al., 2022).

### 1.2 Qigong and its benefits

Qigong is an oriental system of mind-body practices aimed at promoting health which involves the execution of well-coordinated movements, adopting specific positions, and engaging in breathing exercises and meditation (Dorcas and Yung, 2003). The term “qigong,” literally translated as “cultivating Qi” or “mastering Qi,” refers to the use of this activity in the context of Traditional Chinese Medicine to improve psychophysical wellbeing and the body's vital energy (“Qi”) (Liu and Chen, 2010). Qigong's history dates back thousands of years, possibly originating with the legendary Huangdi, the Yellow Emperor, and the medical books attributed to him (Ng, 1999). While its exact origins remain uncertain, this practice stands as one of the oldest roots of Chinese culture: even today, qigong remains widely practiced in the general population and it is not uncommon to see individuals doing qigong exercises in public parks of major Chinese cities (Palmer, 2003).

The various forms of qigong, influenced by Eastern religious traditions (Taoism, Buddhism, Confucianism) and martial arts, are sometimes grouped into two major categories: active qigong, which is more dynamic, and passive qigong, characterized by a predominant meditative component (Klich and Milert, 2018). Active forms involve more movement, dynamism, and higher physical engagement, whereas passive qigong forms are more static, focusing on maintaining specific postures, breath control exercises, and meditative practices. Nevertheless, in qigong sessions, there is a common practice of incorporating a harmonious blend of both active and passive forms, with the choice between the two being guided by the individual's intentions and current physical fitness level (this practice is accessible to individuals of all ages, even suffering from chronic conditions) (Tsang et al., 2002).

Modern science, based on studies conducted in recent years, attributes the following benefits to qigong practice:

- Promotion of psychophysical relaxation with better stress and emotion management (particularly useful for the working-age population) (Wang et al., 2014; van Dam, 2020).
- Improvement of respiratory kinetics, strengthening the diaphragm, and thoracic muscles (Xu et al., 2022).
- Reduction of falls and increase in skeletal mineral density, enhancing overall quality of life and functionality related to daily needs (significant for the elderly) (Rogers et al., 2009; Song et al., 2017).
- Potential contribution to reducing blood pressure in hypertensive subjects and improving pain control in individuals with painful symptoms related to various chronic pathologies (Bai et al., 2015; Ching et al., 2021).
- Possible improvement in inflammatory indices and immune system functionality, likely indirectly attributed to the anti-stress effect (Półrola et al., 2018; Oh et al., 2020).

Overall, qigong practice implies a holistic approach that targets both the physical and mental aspects of health (Kurt et al., 2022), offering potential benefits for mitigating specific symptoms like chronic fatigue, cognitive impairment, and mood disturbances in Long-COVID-19 recovery. Three elements merit specific attention:

- Movement: qigong involves gentle, flowing movements that promote flexibility, balance, and circulation throughout the body. These movements can help alleviate chronic fatigue by boosting overall vitality (McGregor et al., 2024). Moreover, the rhythmic nature of qigong movements induces a relaxation response in the body, reducing tension and promoting a sense of calmness that may contribute to improved mood and cognitive function (Zhang Q. et al., 2023).
- Breath control: qigong emphasizes deep, diaphragmatic breathing techniques that synchronize breath with movement. Controlled breathing exercises can help regulate the autonomic nervous system, promoting relaxation and reducing stress levels (Zaccaro et al., 2018). By enhancing oxygenation of tissues and improving respiratory function, qigong may alleviate symptoms of fatigue and enhance cognitive clarity. Additionally, mindful breathing practices in qigong can cultivate present-moment awareness and mindfulness, which are associated with improved mood and cognitive function (Johnson et al., 2015).
- Meditation: qigong incorporates meditation techniques that cultivate mental focus, clarity, and emotional balance. Through meditation, practitioners learn to observe their thoughts and emotions without judgment, promoting a sense of inner peace and emotional resilience (Wang et al., 2016). This can help alleviate mood disturbances such as anxiety and depression (Saeed et al., 2010) commonly associated with Long-COVID-19. Additionally, meditation practices in qigong may enhance cognitive function by improving attention, concentration, and memory recall.

In light of these considerations and the recognized benefits of physical therapies in accelerating the recovery of patients afflicted by COVID-19 or experiencing persistent manifestations of the disease (Pollini et al., 2024), qigong has been integrated into strategies aimed at enhancing individual health during the pandemic (Feng et al., 2020; Yang et al., 2022; Zhong et al., 2023). However, to date, there is a lack of a systematic overview of the evidence on the subject, capable of defining the efficacy of the treatment in this type of patient. Notably, recent research underscores that qigong constitutes an ideal form of exercise (Feng et al., 2020), particularly pertinent within confined spaces, and feasible for individuals to perform independently with a high degree of safety (Jahnke et al., 2010); furthermore, it requires minimal equipment, thereby promoting cost-effectiveness, and remains accessible to individuals of all genders and age groups (Phansuea et al., 2020). Therefore, conducting a systematic review can be useful to inform future research efforts and optimize the role of qigong in the management and rehabilitation of COVID-19 patients.

### 1.3 Research aim

This review aims at investigating the efficacy of qigong training for COVID-19 integrative support and Long-COVID-19 rehabilitation.

## 2 Materials and methods

This systematic review adhered to the guidelines reported in the PRISMA statement (Liberati et al., 2009). The review protocol, registered in the Open Science Framework (OSF) under the following doi: 10.17605/OSF.IO/7K5X6, was devised in January 2024 and subsequently published online in March 2024 (URL: https://osf.io/7k5x6), following the literature search but preceding the data extraction process.

Incorporated into our analysis were relevant clinical investigations assessing the impact of practicing qigong on recovery from COVID-19 and long-COVID-19 symptoms, with no limitations on publication dates. To ensure robust selection criteria, studies had to be available in the English language or, at the very least, include an English abstract or summary. Furthermore, selected studies had to be formally published in peer-reviewed journals as original research articles.

The subsequent PICOS criteria were applied for article inclusion in this review:

- P (population): patients diagnosed with COVID-19 or Long-COVID-19 syndrome (utilizing qigong for integrative treatment or rehabilitative purposes).
- I (intervention): qigong practiced for any number of sessions, either alone or in combination with other treatments (standard care, drug therapy, nutritional supplements, dietary advice, other types of physical activity, massage, etc.).
- C (comparison): the comparison category encompassed any type, including studies with no control.
- O (outcomes): significant enhancements in symptoms associated with COVID-19 and Long-COVID-19 over the course of time, including improvements in both physical and psychological wellbeing.
- S (study design): clinical investigations comprising controlled trials, observational studies, and case reports. Laboratory experiments conducted *in vitro* or *in vivo* with animal or cell models were intentionally excluded from the primary search.

A systematic screening of PubMed, EMBASE, Web of Science, Scopus, and Google Scholar was carried out to identify relevant studies. The search covered data from the inception of these databases up until February 2024. The search strategies used for each scientific database were the following:

- PubMed: (“qi gong”[Title/Abstract] OR “qi kung”[Title/Abstract] OR “chi gong”[Title/Abstract] OR “chi kung”[Title/Abstract] OR “qigong”[Title/Abstract] OR “chigong”[Title/Abstract]) AND (“COVID-19”[Title/Abstract] OR “COVID19”[Title/Abstract] OR coronavirus[Title/Abstract]).
- EMBASE: (‘qi gong':ab OR ‘qi kung':ab OR ‘chi gong':ab OR ‘chi kung':ab OR ‘qigong':ab OR ‘chigong':ab) AND (‘covid-19':ab OR ‘covid19':ab OR coronavirus:ab).
- Web of Science: (AB=(‘qi gong') OR AB=(‘qi kung') OR AB=(‘chi gong') OR AB=(‘chi kung') OR AB=(‘qigong') OR AB=(‘chigong')) AND (AB=(‘covid-19') OR AB=(‘covid19') OR AB=(coronavirus)).
- Scopus: (TITLE-ABS-KEY (‘qi AND gong') OR TITLE-ABS-KEY (‘qi AND kung') OR TITLE-ABS-KEY (‘chi AND gong') OR TITLE-ABS-KEY (‘chi AND kung') OR TITLE-ABS-KEY (‘qigong') OR TITLE-ABS-KEY (‘chigong')) AND (TITLE-ABS-KEY (‘covid19') OR TITLE-ABS-KEY (‘covid19') OR TITLE-ABS-KEY (coronavirus)).
- Google Scholar (limited to the first 100 results): “Qigong” AND “COVID-19.”

One researcher (M.A.) initially evaluated all materials obtained through the database search, concentrating on titles and abstracts. Subsequently, a second investigator (D.D.) reviewed articles that met the aforementioned inclusion criteria. This two-tiered assessment process was structured to ensure a proper selection of relevant studies for subsequent analysis. Disagreements between the two researchers were resolved through thorough discussion until a consensus was reached, ensuring the accuracy of the final selection. Inter-rater reliability scores were calculated to evaluate agreement between the two researchers: out of the 170 articles screened after duplicates were removed, they concurred on including 10 articles for subsequent full-text analysis, agreed on excluding 155 studies, and for five studies, there were some discussions. This led to an overall agreement of 97%, with a Cohen's κ value of 0.78, indicating substantial agreement. Following the article selection process, one investigator (M.A.) manually compiled information from studies meeting the inclusion criteria using an Excel spreadsheet. Simultaneously, the second researcher (D.D.) conducted a random verification process to validate the accuracy and completeness of the collected data.

The essential data components extracted from the studies included in the review comprised participant demographics, specific research methodologies, relevant details about the intervention and its comparison, as well as the documented outcomes. The key findings from the literature review were summarized and then discussed to generate a qualitative synthesis. Additionally, *p*-values indicating statistically significant differences favoring qigong practice for disease recovery were provided, along with the intervention effect size. In cases where effect size data were unavailable but essential information was present in the original text of the articles, effect sizes were computed using suitable statistical tools tailored for each study design, as outlined here: https://www.psychometrica.de/effect_size.html.

Each eligible Randomized Controlled Trials (RCTs) underwent evaluation utilizing the Jadad scale (Jadad et al., 1996). Each study underwent an assessment resulting in an overall rating between −1 and 5, indicating its methodological quality. Studies scoring 3 or higher were deemed to be of high quality, while those scoring lower were categorized as low quality. Despite the Jadad score criteria stipulating double-blinded trials for high quality, a single-blind design was deemed acceptable due to the inherent difficulty in fully concealing a physical intervention like qigong practice. Non-RCT studies were assessed to ensure compliance with specific guidelines recommended by the EQUATOR network (Simera et al., 2009), including STROBE for observational studies and CARE for case reports. Furthermore, the quality assessment of pre-post studies and case reports was conducted using the NIH - National Institutes of Health (URL: https://www.nhlbi.nih.gov/health-topics/study-quality-assessment-tools) and JBI - The Joanna Briggs Institute (URL: https://jbi.global/critical-appraisal-tools) tools, respectively. The risk-of-bias assessment was used to inform the review discussion.

## 3 Results

After screening all databases, nine studies were eventually deemed eligible for inclusion (Chen et al., 2020; Liu et al., 2021; Tang et al., 2021; Brough et al., 2022; Mekky et al., 2022; Patel et al., 2023; Wang et al., 2023; Xing et al., 2023; Zhang H. et al., 2023). Among them, four were RCTs (Liu et al., 2021; Wang et al., 2023; Xing et al., 2023; Zhang H. et al., 2023), one was a controlled trial with no randomization of the participants (Mekky et al., 2022), while the remaining were three pre-post studies without any control group and a case report (see Table 1 for further details). One study was presented as a conference abstract (Mekky et al., 2022), whereas full-text versions were accessible for the remaining studies.

**Table 1:** Summary of the included studies.

**Main health condition**	**Population (n)**	**Intervention**		**Comparison (n)**	**Mental wellbeing/QoL^†^**	**Sleep quality^†^**	**Respiratory symptoms^†^**	**Study design**	**References**
		**Details**	**Qigong exercises**						
COVID-19	177 patients with asymptomatic COVID-19 Age (range): 18–60 yo Gender: 111 M/66 F	Standard care + Music therapy + Qigong training (60 min/day) for the entire quarantine period (6–7 days on average) (*n =* 96)	Baduanjin (Eight Pieces of Brocade)	Standard care (*n =* 81)	✓	✓	-	Randomized controlled trial	Wang et al., 2023
	154 patients with asymptomatic COVID-19 Age (range): 18–65 yo Gender: 64 M/90 F	Standard care + Health education + Qigong training (20 min/day) for 12 days (*n =* 74)	11 exercises from the Yi Jin Jing method	Standard care + Health education (*n =* 80)	✓	-	-	Randomized controlled trial	Xing et al., 2023
	128 patients hospitalized for severe COVID-19 Age (range): 20–80 yo Gender: 55 M/73 F	Standard care + Acupressure + Qigong training (40 min/day) for the entire hospitalization period (18–21 days on average) (*n =* 64)	Liu Zi Jue (Six Healing Sounds)	Standard care (*n =* 64)	-	-	✓	Randomized controlled trial	Liu et al., 2021
	40 patients with COVID-19 Age (mean ± SD): 41.30 ± 7.73 yo - intervention group, 42.10 ± 8.47 yo - control group Gender: 24 M/16 F	Standard care + Music therapy + Qigong training (60 min/day) for the entire quarantine period (?) (*n =* 20)	Baduanjin (Eight Pieces of Brocade)	Standard care (*n =* 20)	✓	✓	-	Randomized controlled trial	Zhang H. et al., 2023
	10 patients hospitalized for non-severe COVID-19 Age (mean ± SD): 50.20 ± 12.04 yo Gender: 6 M/4 F	Standard care + Qigong training (30 min/day) for 2 weeks (*n =* 10)	Eight-segment pulmonary rehabilitation exercises	None	✓	-	✓	Pre-post study with no control group	Chen et al., 2020
Long-COVID-19	357 patients with insomnia after COVID-19 Age: 18 + yo Gender: M/F?	Qigong training (? min/day) for 2 weeks + Z-drugs/sleep medications (*n =* 106)	?	Z-drugs/sleep medications (*n =* 251)	-	✓	-	Controlled study with no randomization	Mekky et al., 2022
	33 patients discharged from hospital after COVID-19 with persistent respiratory symptoms Age (mean ± SD): 43.2 ± 10.4 yo Gender: 17 M/16 F	Qigong training (20 min/day) for 4 weeks (*n =* 33)	Liu Zi Jue (Six Healing Sounds)	None	-	-	✓	Pre-post study with no control group	Tang et al., 2021
	25 patients with Long-COVID-19 Age: 15 + yo (most of the participants (20/25) had an age comprised between 25 and 65 yo) Gender: 7 M/15 F/3 ?	A program including Qigong training, nutritional support, meditation exercises, group discussions, and aromatherapy for 4 weeks (*n =* 25)	?	None	✓	-	-	Pre-post study with no control group	Brough et al., 2022
	1 patient with Long-COVID-19 respiratory, gastrointestinal, and systemic symptoms Age: 62 yo Gender: 1 F	Taichi exercises for 2 months after the acute phase of COVID-19, then Qigong (10 min/day) + Taichi (35 min/day) training every day for as long as the symptoms persisted (*n =* 1)	Gentle exercises with a focus on controlled breathing	None	-	-	✓	Case report	Patel et al., 2023

F, Females; M, Males; yo, years old. ^†^Significant improvements (✓) in mental wellbeing/QoL (Quality of Life), sleep quality, and/or respiratory symptoms associated with the intervention. ? stands for unavailable/undisclosed information.

**Figure 1:**
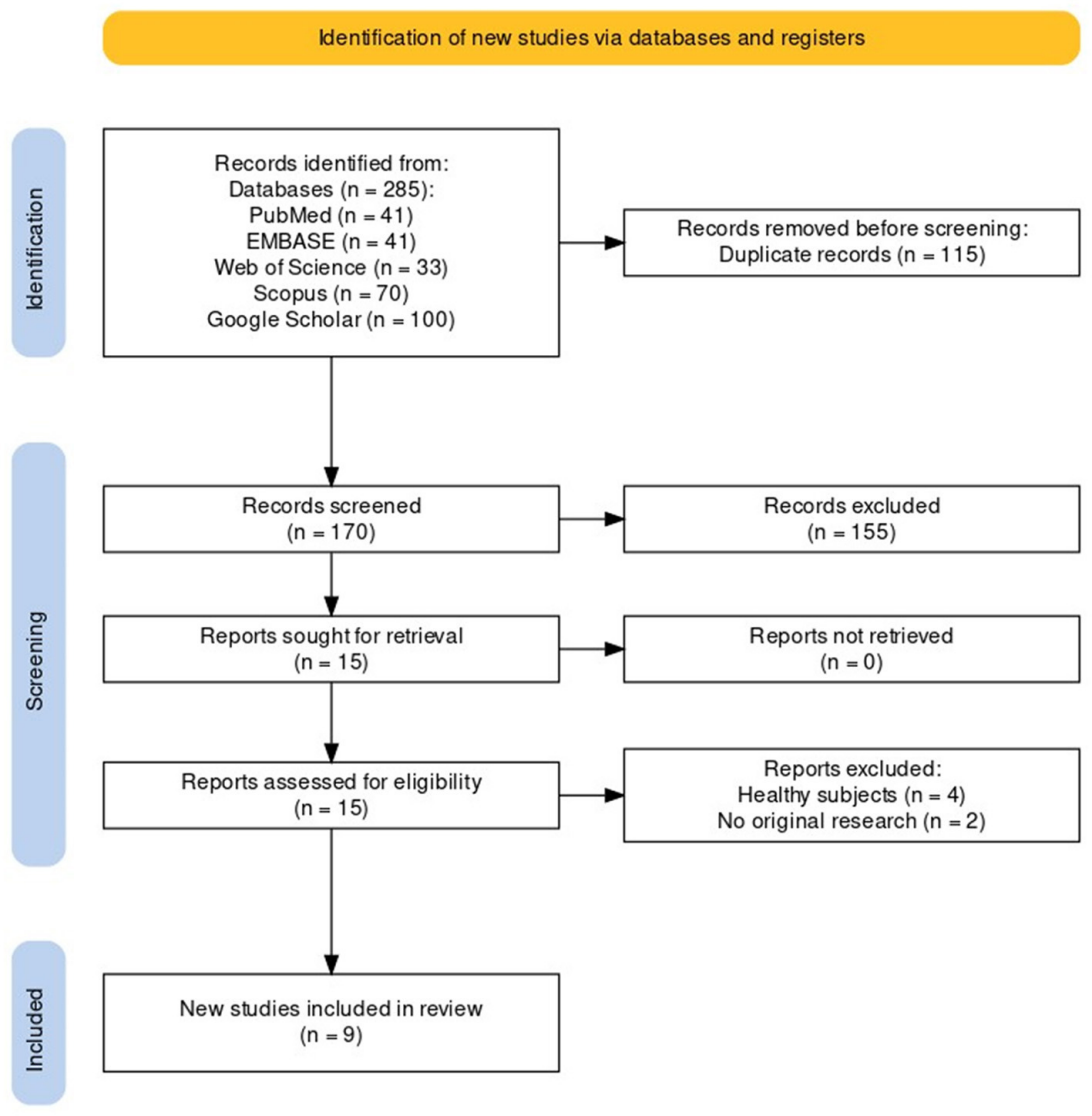
PRISMA flowchart describing the article selection process (Haddaway et al., 2022).

The article selection process is summarized in a dedicated flowchart (Figure 1), as requested by the PRISMA recommendations. A total of 285 research items were screened, comprising 41 from PubMed, 41 from EMBASE, 33 from Web of Science, 70 from Scopus, and 100 from Google Scholar. Following an initial screening, 115 duplicates were eliminated, and subsequently, 155 records were excluded based on title and abstract. Fifteen studies underwent full-text analysis, of which six were excluded either due to the absence of patients with COVID-19 or Long-COVID-19 symptoms (Li et al., 2022; Yang et al., 2022; Gonçalves et al., 2023; Seiça et al., 2023) or because they were not original research articles but rather narrative literature reviews (Feng et al., 2020; Mendo et al., 2022). Ultimately, nine studies met the criteria for inclusion in this review.

Five of the included trials examined the integrative use of qigong in COVID-19 treatment (Chen et al., 2020; Liu et al., 2021; Wang et al., 2023; Xing et al., 2023; Zhang H. et al., 2023), while four studies delved into its efficacy in enhancing the recovery of Long-COVID-19 patients (Tang et al., 2021; Brough et al., 2022; Mekky et al., 2022; Patel et al., 2023). Within the former group of trials, patient numbers varied from a minimum of 10 to a maximum of 177 (median: 128), with all but one study (Chen et al., 2020) adopting a randomized controlled design. Qigong training, practiced for 20–60 min daily throughout the COVID-19 duration, was complemented by standard medical care and other integrative therapies such as acupressure, music therapy, and health education (see Table 1 for additional information). Conversely, controlled groups, if included, solely received standard treatment as per current medical guidelines. Regardless of the severity of COVID-19, individuals undergoing qigong practice demonstrated superior physical and psychological outcomes, including improvements in respiratory symptoms, anxiety levels, sleep quality, mental wellbeing, and health-related quality of life (see Table 2 for further details). Additionally, four studies explored the effects of qigong training (Tang et al., 2021; Brough et al., 2022; Mekky et al., 2022; Patel et al., 2023), either independently or in conjunction with other treatments, on Long-COVID-19 symptoms, encompassing persistent respiratory dysfunction, dizziness, sleep disturbances, and compromised health-related quality of life. Across these studies, which comprised sample sizes ranging from 1 to 357 (median: 29), improvements in the measured outcomes were consistently observed, with some instances demonstrating additional benefits related to weight management. Among the qigong exercises and forms, the most commonly practiced by the study participants were Liu Zi Jue (Six Healing Sounds) and Baduanjin (Eight Pieces of Brocade).

**Table 2:** Main outcomes and results of the studies eligible for inclusion.

**Main health condition**	**Outcomes**	**Statistical significance (*p* value)**	**Effect size**	**Study design**	**References**
COVID-19	↑ Mental wellbeing and health-related QoL: GAD-7 (mean difference, 95% CI): 2.9 [2.2; 3.6]^*^; PHQ-9 (mean difference, 95% CI): 3.6 [2.9; 4.4]^*^; PHCS (mean difference, 95% CI): −5.0 [−6.1; −3.9]^*^↑ Sleep quality: JSS (mean difference, 95% CI): 2.7 [2.0; 3.3]^*^	GAD-7: *p < * 0.001 PHQ-9: *p < * 0.001 PHCS: *p < * 0.001 JSS: *p < * 0.001	d (GAD-7) = 0.70 d (PHQ-9) = 0.35 d (PHCS) = 0.28 d (JSS) = 0.32	RCT	Wang et al., 2023
	↓ Anxiety: HAMA total score (mean ± SD): 2.50 ± 5.05 vs. 4.71 ± 7.07^*^ Significant changes were also reported for the HAMA mental and somatic scores	HAMA total score: *p =* 0.028	d (HAMA) = 0.36	RCT	Xing et al., 2023
	↓ Dyspnea: mMRC (mean difference, 95% CI): −0.3 [−0.6; 0.1]^*^; MBS (mean difference, 95% CI): −0.7 [−1.3; 0.0]^*^ ↓ Days of hospitalization: 18.5 vs. 20.8^*^↓ Cough (days of symptom duration): 14.3 vs. 17.0^*^	mMRC: *p =* 0.018 MBS: *p =* 0.045 Days of hospitalization: *p =* 0.042 Cough duration: *p =* 0.046	d (mMRC) = 0.19 d (MBS) = 0.52 d (days of hospitalization) = 0.37 d (cough duration) = 0.35	RCT	Liu et al., 2021
	↓ Anxiety: SAS (mean ± SD): 44.44 ± 5.85 vs. 48.03 ± 3.23^*^↑ Mood: SDS (mean ± SD): 46.73 ± 7.04 vs. 51.84 ± 5.73^*^↑ Sleep quality: PSQI (mean ± SD): 1.10 ± 0.71 vs. 1.65 ± 0.81^*^	SAS: *p =* 0.023 SDS: *p =* 0.016 PSQI (sleep quality): *p =* 0.029	d (SAS) = 0.76 d (SDS) = 0.80 d (PSQI) = 0.72	RCT	Zhang H. et al., 2023
	↑ Physical activity, perception of dyspnea, and QoL (no numerical findings)	/	/	Pre-post study	Chen et al., 2020
Long-Covid-19	↑ Sleep quality: improvement among cases (%): 68.18% vs. controls: 64.20%	Not reported	RR = 0.89	CS	Mekky et al., 2022
	↑ Functional capacity (MIP, PIF, DM-DB) and QoL (HAMA and SF-36) MIF (mean increase ± SD): 13.46 ± 20.06 cmH2O^*^ PIF (mean increase ± SD): 0.74 ± 0.58 L/sec^*^ DM-DB (mean increase ± SD): 0.57 ± 1.18^*^	MIF: *p < * 0.001 PIF: *p < * 0.001 DM-DB: *p =* 0.009	Pre- and post-test data graphically reported	Pre-post study	Tang et al., 2021
	↑ Health-related QoL: WHHQ-18 (mean ± SD): from 33.7 ± 12.5 at baseline to 39.5 ± 10.8 at the end of the treatment	Not reported	d (QoL) = 0.50	Pre-post study	Brough et al., 2022
	↓ Dyspnea and improvement in SpO2 levels from 83% to 93% ↓ Dizziness ↑ Balance ↑ Weight control	/	/	Case report	Patel et al., 2023

CS, Controlled Study with no randomization of the participants; d, Cohen's d (measure of effect size); DM-DB, Diaphragm Movement in Deep Breathing; GAD-7, General Anxiety Disorder (7 items); HAMA, Hamilton Anxiety Rating Scale; JSS, Jenkins Sleep Scale; MBS, Modified Borg Dyspnea Scale; MIP, Maximal Inspiratory Pressure; mMRC, Modified British Medical Research Council Questionnaire (Dyspnea Scale); PHCS, Perceived Health Competence Scale; PHQ-9, Patient Health Questionnaire (9 items); PIF, Peak Inspiratory Flow; PSQI, Pittsburgh Sleep Quality Index; QoL, Quality of Life; RCT, Randomized Controlled Trial; RR, Risk Ratio; SAS, Self-rating Anxiety Scale; SD, Standard Deviation; SDS, Self-rating Depression Scale; SF-36, Short Form Health Survey (36 items); SpO2, Oxygen Saturation; WHHQ-18, Warwick Holistic Health Questionnaire (18 items). ^*^p < 0.05 (significant change in favor of the intervention).

Overall, this review encompassed four RCTs (Liu et al., 2021; Wang et al., 2023; Xing et al., 2023; Zhang H. et al., 2023), which primarily investigated the efficacy of qigong in COVID-19 integrative care, emphasizing its advantageous effects on psychological wellbeing. Conversely, non-RCT studies (Chen et al., 2020; Tang et al., 2021; Brough et al., 2022; Mekky et al., 2022) included in this review primarily focused on qigong practice for Long-COVID-19 management, assessing benefits across both physical and psychological domains. Another distinction between RCTs and non-RCTs lies in the administration of qigong: in RCTs, qigong was predominantly administered alongside other therapies, whereas in non-RCTs, it was often administered alone and occasionally compared with no treatment. Furthermore, insights from the sole case report incorporated in this review indicate that qigong may offer benefits beyond respiratory enhancements, extending to weight management during the recovery phase from Long-COVID-19 (Patel et al., 2023).

The RCTs included in the review (Liu et al., 2021; Wang et al., 2023; Xing et al., 2023; Zhang H. et al., 2023) consistently demonstrated high quality. However, some methodological weaknesses were identified, particularly in terms of inadequate blinding of study investigators and notable dropout rates in one instance (further details in the Supplementary material). In contrast, non-RCTs lacked clear adherence to internationally recognized methodological guidelines in all cases. One study could not be evaluated due to its reporting as a conference abstract (Mekky et al., 2022). Pre-post studies examined in this review exhibited an average-to-low quality, characterized by limited sample sizes and, in some cases, insufficient information on statistical analysis or study design (see the Supplementary material for further specifics). However, the sole case report included in this review achieved a high score in the NIH quality assessment.

## 4 Discussion

### 4.1 Critical overview of the available evidence and mechanisms of action

The review included both trials investigating the use of qigong in COVID-19 integrative treatment and studies examining its effectiveness in Long-COVID-19 recovery (see Table 1). Qigong, practiced daily for 20–60 min alongside standard medical care, not only led to superior psychological and physical outcomes in COVID-19 patients but also contributed to shortening the duration of the disease (Liu et al., 2021). Similarly, qigong showed promising results in alleviating Long-COVID-19 symptoms, with improvements observed across various health indicators. In general, psychological benefits were supported by stronger evidence. All the same, the intervention primarily yielded effect sizes ranging from 0.25 to 0.75, indicative of a moderate impact on the outcomes measured: this suggests that qigong had a noticeable impact on the outcomes measured in the studies, with effects falling somewhere between small and large (see Table 2 for additional details). Such a medium effect size underscores the meaningfulness of the intervention's impact on the variables under consideration, further supporting its efficacy in producing tangible results. The most commonly practiced qigong exercises were Liu Zi Jue and Baduanjin.

Interpreting the effects of qigong in light of neuroscientific theories such as the polyvagal theory suggests that the benefits of this practice on psychophysical relaxation result from stimulation induced by exercise on the autonomic nervous system's regulatory centers, particularly the vagal tone, promoting ventral vagal complex activity (Seppala et al., 2017). This could lead to increased control and resilience of the autonomic nervous system responses to external stressors, enhancing cardiovascular and respiratory activity, mood tone, as well as a general effect of increased concentration and psychophysical relaxation (Donelli et al., 2023). Thus, the traditional concept of qigong as a practice restoring “Qi” balance can be interpreted neuroscientifically as the potential to contribute, through exercises, to “harmonizing” the activity of both the ventral and dorsal vagal complexes, whose physiological actions are mutually antagonistic, ultimately promoting individual psychophysical wellbeing. Qigong, as a training to achieve a state of “inner silence,” is a discipline that requires simultaneous focus and relaxation during movement: this implies a simultaneous presence of high vagal tone and orthosympathetic tone, which is maximally beneficial because it enhances control over heart rate, breathing, and phonation, and also induces beneficial eustress for immune and biohumoral reasons (Donelli et al., 2023). Indeed, this interpretative paradigm has been applied with interest and scientific credibility to other meditative disciplines, such as Yoga, originating from the Ayurvedic tradition (Sullivan et al., 2018), and mindfulness-based techniques (Lucas et al., 2018; Poli et al., 2021).

The potential effectiveness of qigong in enhancing respiratory function and improving psychological outcomes, as evidenced in the included studies (refer to Table 2 for further details), can be attributed to several underlying mechanisms. Firstly, the controlled breathing exercises inherent in qigong practice can promote respiratory muscle strength and endurance, thereby improving lung capacity and efficiency (Ding et al., 2014). Additionally, the rhythmic breathing patterns facilitate relaxation and stress reduction, which can alleviate anxiety and improve overall psychological wellbeing (Ng and Tsang, 2009). Moreover, the gentle movements involved in qigong promote circulation and oxygenation of tissues, which may aid in tissue repair and enhance overall respiratory health (Lee et al., 2003). Furthermore, the meditative aspect of qigong fosters mindfulness and emotional regulation, helping individuals cope with stress, anxiety, and depression (Chow et al., 2012; Yeung et al., 2018). These combined physiological and psychological benefits make qigong a promising adjunctive therapy for improving both respiratory function and mental health outcomes.

From a scientific perspective, evidence-based rehabilitative protocols must include effective strategies addressing four fundamental points (Osadnik et al., 2015; Pt and Ccc-A/slp, 2015; Stott and Quinn, 2017; Nicolau et al., 2022):

- Exercise, including any motor activity stimulating the patient's functional reserve following an acute illness.
- Practice, involving the repetition of exercises for a sufficient time to achieve the maximum possible degree of functional recovery.
- Psychological support, beneficial for the individual's mental wellbeing.
- Patient education, facilitating proper self-management of health conditions and attentive long-term monitoring.

In general, qigong aligns well with the four fundamental points of evidence-based rehabilitative protocols. It involves a series of slow, controlled movements and postures, providing a form of motor activity that stimulates the body's functional reserve following illness, promoting physical rehabilitation after an acute illness. By emphasizing the repetition of exercises over time, qigong supports maximum functional recovery and enhances neuroplasticity, proprioceptive re-education, cardio-respiratory kinetics, and muscle tone. This aspect assumes particular significance given the respiratory manifestations and sequelae often associated with COVID-19 (Boutou et al., 2021): in fact, by incorporating techniques aimed at enhancing lung capacity, improving oxygenation, and promoting respiratory muscle strength, qigong breathing exercises can mitigate the respiratory challenges posed by COVID-19 and its long-term complications. Moreover, qigong incorporates mindfulness techniques and focused breathing, which can have a profound impact on mental wellbeing, offering valuable psychological support during the recovery process and promoting reintegration into the social context. This holds significance, particularly concerning the cognitive impairment associated with Long-COVID-19, commonly referred to as “brain fog” (Nouraeinejad, 2023): cognitive exercises have shown efficacy in addressing this condition (Rabaiotti et al., 2023), and research indicates that qigong can enhance cognitive functions, particularly in elderly populations (Qi et al., 2021). Through qigong practice, individuals can learn self-management strategies for their health conditions, including proper breathing techniques, body awareness, and stress management, empowering them to take an active role in their recovery and long-term health journey. Additionally, qigong instructors can offer educational resources and support to help patients navigate their rehabilitation effectively. Qigong is optimal for the elderly as it constitutes a gentle yet comprehensive exercise, engaging aspects such as respiration, cardiac function, resistance training, balance, and coordination. However, for younger age groups, it may be more beneficial for neuro-autonomic aspects, albeit less effective in achieving cardiovascular prevention goals due to its insufficient intensity to count toward the recommended amount of weekly aerobic activity (at least 150 min/week) (Pelliccia et al., 2021).

Qigong is a cost-effective and accessible health-promoting practice, demonstrating versatility in its application across various demographics, even during the COVID-19 pandemic (Jahnke et al., 2010; Klich and Milert, 2018; Mendo et al., 2022). Its simplicity makes it attainable for individuals with severe illnesses, chronic conditions, or disabilities, ensuring inclusivity in its practice (Ng and Tsang, 2009; Guo et al., 2018). Furthermore, its adaptability allows for indoor sessions, making it feasible even within the confines of small living spaces like apartments and quarantine rooms, while its compatibility with outdoor environments encourages practitioners to engage with nature in parks or forests (Feng et al., 2020; Hung et al., 2021). Additionally, the digital age has facilitated the dissemination of qigong teachings through online platforms, enabling individuals to participate in live or recorded training sessions from the comfort of their homes (Oh et al., 2021; Akinci et al., 2022). This multifaceted accessibility underscores the potential of qigong as a widely accessible and sustainable means of promoting physical and mental wellbeing among patients with COVID-19 symptoms or long-term sequelae.

Furthermore, qigong's compatibility with various treatment modalities and practices makes it a versatile addition to integrated therapeutic regimens, enhancing its efficacy in addressing diverse health needs and complementing medical interventions. Qigong's potential to promote relaxation and reduce stress may offer additional benefits, potentially leading to a reduction in the intake of anxiolytic drugs or sleep inducers (Wang et al., 2013; Mekky et al., 2022; Xing et al., 2023; Zhang H. et al., 2023). Moreover, regular engagement in qigong can cultivate a deeper appreciation for an active lifestyle among practitioners, encouraging them to embrace physical activity as an integral part of their daily routine (Horowitz, 2009). Indeed, in a case report, the overweight individual with Long-COVID-19 symptoms who commenced qigong training successfully achieved better long-term weight management (Patel et al., 2023).

Finally, research suggests that regular practice of qigong can effectively reduce stress, anxiety, and depressive symptoms by promoting relaxation, enhancing emotional regulation, and fostering a sense of inner calm (Wang et al., 2013). Its emphasis on mindful breathing and slow, deliberate movements helps individuals to connect with their bodies, quiet the mind, and cultivate resilience in the face of adversity. Particularly in professions like healthcare, where workers are often exposed to high levels of stress and emotional strain, integrating qigong into daily routines can offer a valuable means of self-care and support mental wellbeing (Gutierrez et al., 2023; Seiça et al., 2023). Similarly, students facing academic pressure and uncertainty can benefit from incorporating qigong practices into their study breaks or daily routines to alleviate stress and improve focus (Li et al., 2022; Gonçalves et al., 2023). In fact, research shows that physical activity (along with socio-economic status) significantly influenced psychological wellbeing and sleep quality in college students during the COVID-19 pandemic (Rassolnia and Nobari, 2024). By providing a holistic approach to mental wellness, qigong offers a valuable resource for individuals navigating challenging circumstances and seeking to enhance their psychological resilience.

### 4.2 Study limitations

One limitation of this review is the potential confounding effect of combining qigong with other therapies in several of the included trials. The co-administration of qigong with additional interventions, such as music therapy or acupressure, makes it challenging to isolate the specific efficacy of qigong alone. Consequently, the observed effects may be influenced by the concurrent treatments, complicating the interpretation of results and making it difficult to attribute improvements solely to qigong practice. Moreover, variations in the duration, frequency, and intensity of qigong sessions across studies could introduce heterogeneity into the results, impacting the ability to draw definitive conclusions about its efficacy. Additionally, the quality of some included studies may be limited, potentially affecting the reliability and generalizability of the findings. Finally, publication bias could influence the results if studies with negative or neutral findings are underrepresented in the literature. These limitations emphasize the need for future well-designed controlled trials with rigorous methodologies to provide more conclusive evidence regarding the isolated efficacy of qigong in COVID-19 and Long-COVID-19 recovery.

As highlighted in a particular trial (Liu et al., 2021), achieving an optimal sample size is crucial for detecting significant physiological effects, if any, of qigong in COVID-19 recovery: in particular, the study suggests that a sample size of approximately 65 participants per trial arm would be necessary to effectively discern any physiological benefits of qigong practice in individuals recovering from COVID-19. Similarly, this sample size would also be applicable in evaluating the effects of qigong on psychological wellbeing.

## 5 Conclusions

In conclusion, while this review highlights the potential benefits of qigong in promoting health and aiding in the recovery of COVID-19 patients, there is a clear need for future studies to further elucidate its therapeutic effects. Rigorous study designs, such as randomized controlled trials with sufficient sample sizes, are necessary to accurately quantify the efficacy of qigong training. Moreover, future research endeavors should prioritize clarity and relevance in research questions, focusing on precision in study design and objectives rather than solely on sample size magnitude. Additionally, investigating the impact of qigong as a standalone intervention, independent of other complementary therapies, can provide insights into its unique contributions to specific health outcomes. By adhering to stringent methodological standards and exploring the effects of qigong in isolation, researchers can advance our understanding of its therapeutic potential and explore its broader integration into clinical practice.

Overall, the available evidence indicates that qigong practice is beneficial for both COVID-19 and Long-COVID-19 recovery, with recommended durations ranging from several days to weeks. A minimum of 20 min per day, up to a maximum of 60 min, is deemed necessary for optimal therapeutic effects. For COVID-19 recovery, even a week of regular practice may yield noticeable benefits, while for Long-COVID-19, it is recommended to continue for at least 1 month to experience more substantial improvements. Key qigong forms such as Liu Zi Jue and Baduanjin, or any gentle total body activity combined with breathing exercises, are particularly effective in promoting physical and psychological wellbeing during recovery from both acute and prolonged phases of the disease.

## Data availability statement

The original contributions presented in the study are included in the article/Supplementary material, further inquiries can be directed to the corresponding author.

## Author contributions

MA: Conceptualization, Data curation, Formal analysis, Funding acquisition, Investigation, Methodology, Project administration, Resources, Software, Supervision, Validation, Visualization, Writing—original draft, Writing—review & editing. DD: Data curation, Methodology, Supervision, Validation, Writing—original draft, Writing—review & editing.
