# Supplementary material for: Evaluating qigong as integrative support for COVID-19 and Long-COVID-19 rehabilitation: a systematic review
Source: Front Psychol. 2024 May 14;15:1403130. doi: 10.3389/fpsyg.2024.1403130 (PMC11130475; doi:10.3389/fpsyg.2024.1403130)
Supplement: Supplementary file 1 [file Data_Sheet_1.docx]

**SUPPLEMENTARY MATERIALS OF “Evaluating Qigong as Integrative Support for COVID-19 and Long-COVID-19 Rehabilitation: A Systematic Review” (2024)**

**Michele Antonelli and Davide Donelli**

**Supplementary Table A**. Quality of the available Randomized Controlled Trials.

| **Item 1** | **Item 2** | **Item 3** | **Item 4** | **Item 5** | **Overall** | **Reference** |
| --- | --- | --- | --- | --- | --- | --- |
| 1 | 1 | 1 | 1 | 0 | 4/5 | [(Wang *et al.*, 2023)](https://paperpile.com/c/FoATNr/DnzjE) |
| 1 | 1 | 0 | 0 | 1 | 3/5 | [(Xing *et al.*, 2023)](https://paperpile.com/c/FoATNr/rMCo2) |
| 1 | 1 | 0 | 0 | 1 | 3/5 | [(Liu *et al.*, 2021)](https://paperpile.com/c/FoATNr/nwvE) |
| 1 | 1 | 1 | 0 | 1 | 4/5 | [(Zhang *et al.*, 2023)](https://paperpile.com/c/FoATNr/43PLf) |

Legends (Jadad scale items):

1. Randomization
2. Method of random sequence generation
3. Blinding
4. Method of blinding
5. Withdrawals and dropouts

**Supplementary Table B**. Quality of the available Pre-Post Studies.

| **1** | **2** | **3** | **4** | **5** | **6** | **7** | **8** | **9** | **10** | **11** | **12** | **Overall** | **Reference** |
| --- | --- | --- | --- | --- | --- | --- | --- | --- | --- | --- | --- | --- | --- |
| Yes | Yes | Yes | NR | NR | Yes | Yes | NR | Yes | NR | NR | NR | 6/12 | [(Chen *et al.*, 2020)](https://paperpile.com/c/FoATNr/hf8a) |
| Yes | Yes | Yes | No | NR | Yes | Yes | NR | Yes | Yes | No | NR | 7/12 | [(Tang *et al.*, 2021)](https://paperpile.com/c/FoATNr/rXkU) |
| Yes | Yes | Yes | No | NR | Yes | Yes | NR | Yes | Yes | Yes | NR | 8/12 | [(Brough, Abel and Priddle, 2022)](https://paperpile.com/c/FoATNr/IEbx) |

Legends (NIH quality assessment items):

1. Was the study question or objective clearly stated?

2. Were eligibility/selection criteria for the study population prespecified and clearly described?

3. Were the participants in the study representative of those who would be eligible for the test/service/intervention in the general or clinical population of interest?

4. Were all eligible participants that met the prespecified entry criteria enrolled?

5. Was the sample size sufficiently large to provide confidence in the findings?

6. Was the test/service/intervention clearly described and delivered consistently across the study population?

7. Were the outcome measures prespecified, clearly defined, valid, reliable, and assessed consistently across all study participants?

8. Were the people assessing the outcomes blinded to the participants' exposures/interventions?

9. Was the loss to follow-up after baseline 20% or less? Were those lost to follow-up accounted for in the analysis?

10. Did the statistical methods examine changes in outcome measures from before to after the intervention? Were statistical tests done that provided p values for the pre-to-post changes?

11. Were outcome measures of interest taken multiple times before the intervention and multiple times after the intervention (i.e., did they use an interrupted time-series design)?

12. If the intervention was conducted at a group level (e.g., a whole hospital, a community, etc.) did the statistical analysis take into account the use of individual-level data to determine effects at the group level?

Yes/No/NR=Not Reported

**Supplementary Table C**. Quality of the available Case Report.

| **Item 1** | **Item 2** | **Item 3** | **Item 4** | **Item 5** | **Item 6** | **Item 7** | **Item 8** | **Overall** | **Reference** |
| --- | --- | --- | --- | --- | --- | --- | --- | --- | --- |
| Yes | Yes | Yes | Yes | Yes | Yes | NR | Yes | 7/8 | [(Patel, Tu and Gittner, 2023)](https://paperpile.com/c/FoATNr/LiHD) |

Legends (JBI tool items):

1. Clear description of the patient’s demographic characteristics
2. Clear description of the patient’s history
3. Clear description of the clinical condition analyzed
4. Clear description of the assessment methods
5. Clear description of the treatment procedure
6. Clear description of post-intervention clinical conditions
7. Report of possible adverse events
8. Indication of takeaway lessons

Yes/No/NR=Not Reported

**References**

[Brough, D.N., Abel, S. and Priddle, L. (2022) ‘A service evaluation of a community project combining psychoeducation and mind-body complementary approaches to support those with long covid in the UK’, *European journal of integrative medicine*, 55, p. 102182. Available at: https://doi.org/](http://paperpile.com/b/FoATNr/IEbx)[10.1016/j.eujim.2022.102182](http://dx.doi.org/10.1016/j.eujim.2022.102182)[.](http://paperpile.com/b/FoATNr/IEbx)

[Chen, J.-M. *et al.* (2020) ‘The Application of Eight-Segment Pulmonary Rehabilitation Exercise in People With Coronavirus Disease 2019’, *Frontiers in physiology*, 11, p. 646. Available at: https://doi.org/](http://paperpile.com/b/FoATNr/hf8a)[10.3389/fphys.2020.00646](http://dx.doi.org/10.3389/fphys.2020.00646)[.](http://paperpile.com/b/FoATNr/hf8a)

[Liu, S.-T. *et al.* (2021) ‘Effect of qigong exercise and acupressure rehabilitation program on pulmonary function and respiratory symptoms in patients hospitalized with severe COVID-19: a randomized controlled trial’, *Integrative medicine research*, 10(Suppl), p. 100796. Available at: https://doi.org/](http://paperpile.com/b/FoATNr/nwvE)[10.1016/j.imr.2021.100796](http://dx.doi.org/10.1016/j.imr.2021.100796)[.](http://paperpile.com/b/FoATNr/nwvE)

[Patel, R., Tu, B. and Gittner, L.S. (2023) ‘The use of Tai chi and qigong to mediate long-haul COVID-19 symptoms: Case report’, *Integrative medicine reports*, 2(1), pp. 1–6. Available at: https://doi.org/](http://paperpile.com/b/FoATNr/LiHD)[10.1089/imr.2022.0086](http://dx.doi.org/10.1089/imr.2022.0086)[.](http://paperpile.com/b/FoATNr/LiHD)

[Tang, Y. *et al.* (2021) ‘Liuzijue is a promising exercise option for rehabilitating discharged COVID-19 patients’, *Medicine*, 100(6), p. e24564. Available at: https://doi.org/](http://paperpile.com/b/FoATNr/rXkU)[10.1097/MD.0000000000024564](http://dx.doi.org/10.1097/MD.0000000000024564)[.](http://paperpile.com/b/FoATNr/rXkU)

[Wang, X. *et al.* (2023) ‘The effect of Baduanjin Qigong combined with five-elements music on anxiety and quality of sleep in asymptomatic patients with COVID-19 infection: A randomised controlled trial’, *Heliyon*, 9(8), p. e18962. Available at: https://doi.org/](http://paperpile.com/b/FoATNr/DnzjE)[10.1016/j.heliyon.2023.e18962](http://dx.doi.org/10.1016/j.heliyon.2023.e18962)[.](http://paperpile.com/b/FoATNr/DnzjE)

[Xing, H. *et al.* (2023) ‘易筋经改善新冠肺炎无症状感染者隔离期焦虑情绪的临床观察’, *Journal of acupuncture and tuina science*, 21(4), pp. 285–293. Available at: https://doi.org/](http://paperpile.com/b/FoATNr/rMCo2)[10.1007/s11726-023-1387-z](http://dx.doi.org/10.1007/s11726-023-1387-z)[.](http://paperpile.com/b/FoATNr/rMCo2)

[Zhang, H. *et al.* (2023) ‘Effect of five-elements music therapy combined with Baduanjin qigong on patients with mild COVID-19’, *Hong Kong Journal of Occupational Therapy*, 36(1), pp. 31–38. Available at: https://doi.org/](http://paperpile.com/b/FoATNr/43PLf)[10.1177/15691861231167536](http://dx.doi.org/10.1177/15691861231167536)[.](http://paperpile.com/b/FoATNr/43PLf)
